# Preoperative Low Prealbumin Is Associated With Recurrence in Patients With Stage II/III Gastric Cancer After Laparoscopic D2 Gastrectomy

**DOI:** 10.3389/fsurg.2022.819514

**Published:** 2022-04-01

**Authors:** Chun Gao, Ci Dian Dan Zeng, Yi Xin Tong, Li Zhu, Sheng Zhang

**Affiliations:** Department of Gastrointestinal Surgery, Tongji Hospital, Tongji Medical College, Huazhong University of Science and Technology, Wuhan, China

**Keywords:** gastric cancer, recurrence, risk factor, prealbumin, recurrence-free survival, cancer

## Abstract

**Background:**

Postoperative recurrence is associated with poor prognosis in patients with gastric cancer. This study aimed to explore predictive factors contributing to recurrence in patients with stage II/III gastric cancer after laparoscopic D2 gastrectomy.

**Methods:**

This retrospective study was conducted at a single tertiary referral hospital. Patients diagnosed with gastric cancer who met the inclusion criteria were included in the study. The clinicopathological characteristics of the patients were collected. The patients were divided into recurrence and non-recurrence groups. The predictive factors were investigated using univariate and multivariate analyses.

**Results:**

In total, 462 patients were included. The incidence of recurrence was 26.4% (122/462) in all patients. The most common recurrence pattern was haematogenous recurrence. In the multivariate analysis, the independent predictive factors for recurrence were serum prealbumin level (*p* < 0.001), prognostic nutritional index (*p* = 0.001), carbohydrate antigen 19-9 (CA19-9) (*p* < 0.001), number of lymph node metastases (*p* < 0.001), signet-ring cell carcinoma (*p* = 0.001), tumor deposit (*p* = 0.001), and no/incomplete adjuvant chemotherapy (*p* < 0.001).

**Conclusions:**

Our findings revealed that nutritional status was an independent predictive factor for recurrence in patients with gastric cancer after D2 gastrectomy. We suggest that patients with risk factors for recurrence receive both nutritional support and intense surveillance.

## Introduction

Gastric cancer (GC) is a common malignancy of the gastrointestinal system and remains one of the deadliest cancers worldwide (1). Curative surgery combined with adjuvant chemotherapy (AC) is the standard treatment for patients with advanced gastric cancer (2–4). The overall survival rate of these patients has improved thanks to the hard work of scholars and physicians. However, the recurrence rate remains high. Most patients with gastric cancer experience recurrence within 2 years after surgery, and recurrence has a poor prognosis. This urges us to identify the risk factors and improve long-term survival (5, 6).

Early recurrence of gastric cancer is usually defined as tumor relapse within 12–24 months of gastrectomy (5, 7, 8) increased risk of gastric cancer recurrence is associated with multiple clinicopathological factors such as tumor size, lymph node metastasis, Lauren histologic type, lymphatic invasion, neural invasion, CA19-9 and postoperative chemotherapy (9–12). Researchers have been investigating the potential risk factors for recurrence in patients with gastric cancer after D2 gastrectomy.

Malnutrition has been widely reported as a poor prognostic factor in patients with advanced gastric cancer (13–15). Parameters such as serum albumin, prealbumin (PA), body mass index (BMI), skeletal muscle mass (SMA), and prognostic nutritional index (PNI) are predictive indicators for both prognosis and postoperative complications in patients with gastric cancer (16). In this study, we aimed to identify potential independent predictive factors for gastric cancer recurrence. Additionally, we examined the correlation between preoperative nutritional status and the risk of recurrence.

## Methods

### Study Design and Patients

Data from patients with gastric cancer who underwent laparoscopic gastrectomy with D2 lymphadenectomy according to the Japanese Gastric Cancer Treatment Guidelines (17) were retrospectively collected and analyzed in a single tertiary referral hospital between January 2016 and May 2020. Patients aged > 18 years who met the following inclusion criteria were recruited: (1) histopathologically confirmed diagnosis of gastric cancer, (2) patients underwent operation as laparoscopic gastrectomy with D2 lymph node dissection, and (3) No evidence of distant metastasis.

The exclusion criteria were (1) incomplete records of important clinical or laboratory data, (2) synchronous diagnosis of other malignancies or stage IV gastric cancer, (3) patients already receiving neoadjuvant therapy, and (4) death within 1 month after the operation.

This study was approved by the Institutional Medical Ethics Committee (TJH20190901), and all aspects of this study complied with the 1964 Helsinki Declaration and its later versions. Informed consent was obtained from all patients.

### Data Collection

Data were retrospectively collected from the electronic medical records. The following data were collected for analysis: (1) Demographic characteristics, such as age, sex, body mass index (BMI), history of smoking or alcohol use; (2) Laboratory characteristics such as absolute neutrophil count (ANC), absolute lymphocyte count (ALC), platelet count, hemoglobin, albumin, prealbumin, and serum tumor markers, such as carcinoembryonic antigen (CEA) and CA19-9; (3) Clinical characteristics, such as tumor size, differentiation, invasion depth (T), presence of lymph node metastases (N), tumor-node-metastasis stage (TNM) (4), number of lymph node metastases, signet-ring cells, tumor deposit (TD), vessel carcinoma embolus, and neural invasion; and (4) Perioperative characteristics, such as postoperative complications (according to the Clavien–Dindo criteria) (18) and adjuvant chemotherapy.

### Follow-Up

Patients were regularly followed-up every month in the first 12 months after surgery and then every 3–6 months thereafter. Follow-up investigations included physical examination, blood tests (such as complete blood count, liver function, CEA, CA19-9, et al.), chest radiography, abdominal contrast-enhanced computerized tomography scanning, and annual endoscopic examination.

### Study Endpoints and Definition of Recurrence

The primary endpoint of this study was postoperative recurrence. Tumor recurrence was defined as the diagnosis of a tumor based on radiologic findings with or without biopsies. Recurrence-free survival (RFS) was defined as the time interval between the date of gastrectomy and the date of recurrence, or the last follow-up without recurrence. According to the literature, recurrence patterns were classified as local recurrence (anastomotic recurrence or gastric remnant carcinoma), haematogenous recurrence (hepatic, lymphatic, pulmonary, bone, or other sites of metastatic disease), and peritoneal recurrence. The secondary endpoint was overall survival (OS). OS was defined as the time interval between the date of gastrectomy and the date of death or last follow-up (19, 20).

### Statistical Analyses

All continuous variables were presented as mean (standard deviation, SD)/median (range) and analyzed using Student's *t*-test or Mann–Whitney U test. Categorical variables were reported as whole numbers and percentages and compared using the chi-squared test or Fisher's exact test. The lower limit of the normal reference range (200 mg/L) in our hospital was used to determine the cut-off value of serum prealbumin level as “low” and “normal”. The prognostic nutritional index (PNI) was calculated as follows: 10 × serum albumin (g/dl) + 0.005 × lymphocyte count (per mm^3^) (16). The cut-off value for PNI was determined using the receiver operating characteristic (ROC) curve. The area under the curve (AUC) was 0.651 (95% CI 0.594–0.708, *p* < 0.05). The Youden index was used to determine the cut-off value of the PNI. RFS and OS were analyzed using the Kaplan–Meier method, and differences were assessed using the log-rank test. Univariate logistic regression was used to evaluate potential predictive factors for recurrence. Only factors with a *p* < 0.1 in univariate analysis were included in the final multivariate analysis model. Multivariate logistic regression was used to identify independent predictive factors for recurrence. All *p*-values were two-sided, with a significance level of 0.05. All statistical tests were performed using SPSS version 24.0 (IBM Corp., Armonk, NY, USA).

## Results

### Clinicopathological Characteristics

Between January 2016 and May 2020, 756 patients with gastric cancer who underwent laparoscopic gastrectomy with D2 lymph node dissection were reviewed and considered eligible for this study. Based on the inclusion and exclusion criteria, 462 patients were included in the final analysis. A flow chart of these patients is shown in Supplementary Figure 1, and their clinicopathological parameters are summarized in Tables 1, 2. The mean age of the included patients was 56.3 ± 10.0 comprised of 326 (70.6%) men and 136 (29.4%) women. Moreover, 36.4 and 63.6% of the patients had stage II or III disease, respectively. Grade 1 and 2 postoperative complications occurred in 13.4% (62/462) of the patients.

**Table 1:** The demographic and laboratory characteristics of all patients (*n* = 462).

**Variables**	**All patients (*N* = 462)**	**No recurrence (*n* = 340)**	**Recurrence (*n* = 122)**	***p*-value**
**Demographic**				
Age, mean (SD), y	56.3 ± 10.0	56.1 ± 9.4	57.1 ± 11.4	0.483
Gender, Male, *n* (%)	326 (70.6%)	250 (73.5%)	76 (62.3%)	0.104
Body mass index mean (SD), kg/m^2^	22.5 ± 3.8	22.8 ± 2.7	22.3 ± 4.1	0.895
Smoking				
No	69.7% (322/462)	67.6% (230/340)	75.4% (92/122)	0.384
Yes	30.3% (140/462)	32.4% (110/340)	24.6% (30/122)	
Alcohol				
No	83.1% (384/462)	80.0% (272/340)	91.8% (112/122)	**0.035**
Yes	16.9% (78/462)	20.0% (68/340)	8.2% (10/122)	
**Laboratory**				
Albumin (g/L), mean (SD)	40.6 ± 4.5	41.3 ± 4.4	38.7 ± 4.3	**<0.001**
Prealbumin (mg/L), mean (SD)	229.3 ± 54.4	241.4 ± 48.3	195.6 ± 56.7	**<0.001**
Serum prealbumin				
≥200 mg/L	74.0% (342/462)	88.2% (300/340)	34.4% (42/122)	**<0.001**
<200 mg/L	35.1% (120/462)	11.5% (40/340)	65.6% (80/122)	
Absolute neutrophil count (*10^9^/L), mean (SD)	3.4 ± 1.5	3.3 ± 1.5	3.4 ± 1.4	0.627
Absolute lymphocyte count (*10^9^/L), mean (SD)	1.6 ± 0.6	1.6 ± 0.6	1.6 ± 0.5	0.3122
Hemoglobin (g/L), mean (SD)	120.4 ± 24.7	123.9 ± 23.6	110.7 ± 25.6	**0.001**
Preoperative serum CEA				
Normal (<5 ng/ml)	86.6% (400/462)	90.0% (306/340)	77.0% (94/122)	**0.011**
Elevated (≥5 ng/ml)	13.4% (62/462)	10.0% (34/340)	23.0% (28/122)	
Preoperative serum CA19-9				
Normal (<37 IU/ml)	92.2% (426/462)	96.5% (328/340)	80.3% (98/122)	**<0.001**
Elevated (≥37 IU/ml)	7.8% (36/462)	3.5% (12/340)	19.7% (24/122)	
PNI				
≥45	78.4% (362/462)	85.3% (290/340)	59.0% (72/122)	<0.001
<45	21.6% (100/462)	14.7% (50/340)	41.0% (50/122)	

*For p-value: Boldface type indicates significant difference*.

*SD, standard deviation; CEA, carcinoembryonic antigen; PNI, prognostic nutritional index*.

**Table 2:** The clinical characteristics of all patients (*n* = 462).

**Variables**	**All patients (*N* = 462)**	**No recurrence (*n* = 340)**	**Recurrence (*n* = 122)**	***p*-value**
**Pathological**				
Tumor size (cm), mean (SD)	4.0 ± 2.1	3.8 ± 2.0	4.6 ± 2.3	**0.017**
TNM stage				
II	36.4% (168/462)	58.2% (198/340)	41.8% (142/340)	**0.004**
III	63.6% (294/462)	21.3% (26/122)	78.7% (96/122)	
T stage				
T2-3	52.8% (244/462)	52.9% (180/340)	52.5% (64/122)	0.948
T4	47.2% (218/462)	47.1% (160/340)	47.5% (58/122)	
Number of lymph node metastasis, median (range)	2 (0–27)	2 (0–27)	5 (0–25)	**<0.001**
Tumor differentiation				
High/moderate	17.3% (80/462)	19.4% (66/340)	11.5% (14/122)	0.160
Poor	82.7% (382/462)	80.6% (274/340)	88.5% (108/122)	
Signet-ring cell carcinoma				
No	85.3% (394/462)	88.2% (300/340)	77.0% (94/122)	**0.034**
Yes	14.7% (68/462)	11.8% (40/340)	23.0% (28/122)	
Tumor deposit				
No	89.2% (412/462)	92.4% (314/340)	80.3% (98/122)	**0.010**
Yes	10.8% (50/462)	7.6% (26/340)	19.7% (24/122)	
Vessel carcinoma embolus				
No	84.8% (392/462)	88.8% (302/340)	73.8% (90/122)	**0.005**
Yes	15.2% (70/462)	11.2% (38/340)	26.2% (32/122)	
Neural invasion				
No	88.7% (410/462)	90.6% (308/340)	83.6% (102/122)	0.139
Yes	11.3% (52/462)	9.4% (32/340)	16.4% (20/122)	
**Postoperative**				
Adjuvant chemotherapy				
Yes	49.8% (230/462)	57.6% (196/340)	27.9% (34/122)	**<0.001**
No or incomplete	50.2% (232/462)	42.4% (144/340)	72.1% (88/122)	
Complications				
No	86.6% (400/462)	87.1% (296/340)	83.3% (104/122)	0.122
Grade 1 and 2	13.4% (62/462)	12.9% (44/340)	16.7% (18/122)	
RFS, median (SD), months	17.5 ± 1.2	25.0 ± 1.2	12.2 ± 0.7	**<0.001**

*For p-value: Boldface type indicates significant difference*.

*SD, standard deviation; TNM, tumor-node-metastasis; AC, adjuvant chemotherapy; RFS, recurrence-free survival*.

A total of 26.4% (122/462) of patients experienced recurrence. Compared to patients without recurrence, patients with recurrence had some noticeable clinical features, including elevated levels of tumor markers (serum CEA and CA19-9), larger tumor size (*p* = 0.017), increased number of lymph node metastases (*p* < 0.001), presence of signet-ring cell carcinoma (*p* = 0.034), presence of tumor deposits (*p* = 0.01), and presence of vessel carcinoma embolus (*p* = 0.005).

Notably, patients with recurrence had a poor nutritional status, such as lower albumin (*p* < 0.001) and prealbumin levels (*p* < 0.001), decreased serum hemoglobin (*p* = 0.001), and lower PNI (*p* < 0.001). The majority (72.1%) of patients with recurrence received incomplete or no adjuvant chemotherapy. The median RFS of patients with recurrence was 12.2 months (95% CI, 11.5–12.9), which is significantly shorter than that of patients without recurrence (25.0 months, 95% CI 23.8–26.2). Other information is summarized in Tables 1, 2.

### Patterns of Recurrence in Gastric Cancer

**Figure 1:**
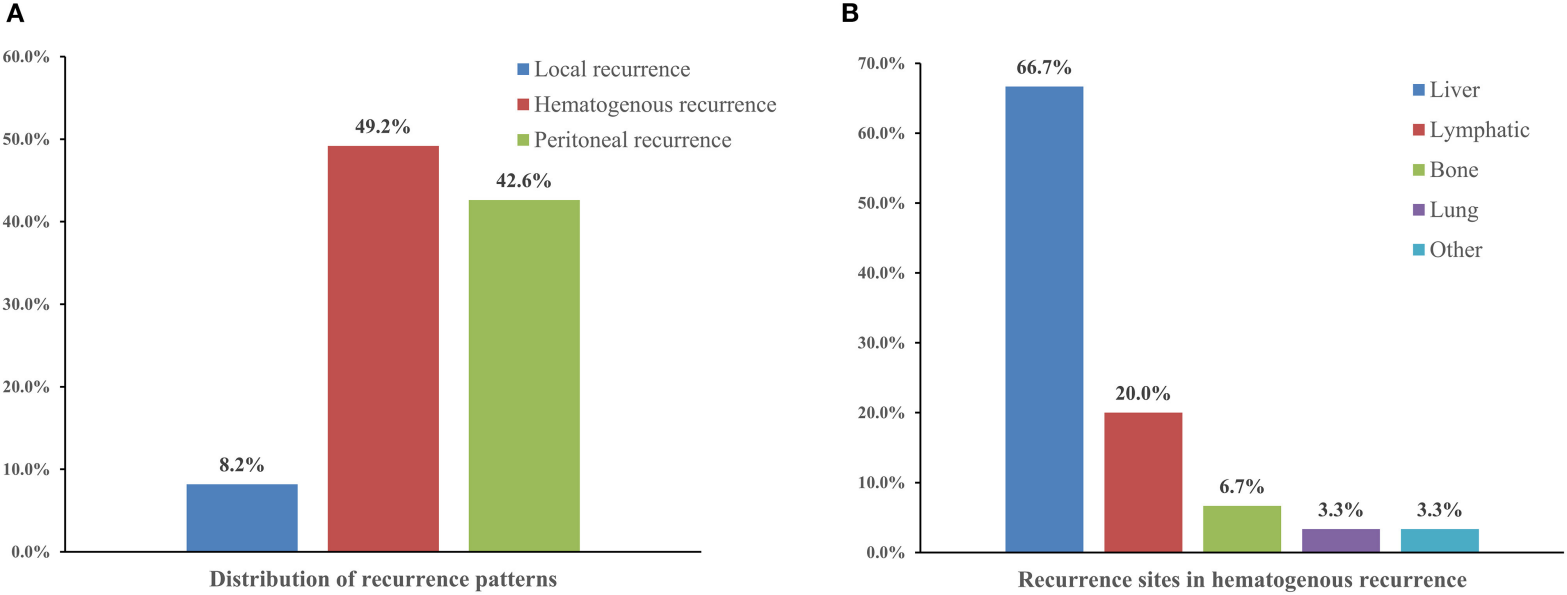
The distribution of recurrence patterns **(A)** and recurrence sites in hematogenous recurrence **(B)** in gastric cancer patients with recurrence.

Of the 112 patients who experienced recurrence, 108 (88.5%) experienced cancer again within 24 months, while the other 14 patients (11.5%) experienced cancer after 24 months. The most frequent pattern of recurrence was haematogenous (49.2%), followed by peritoneal (42.6%) and local (8.2%). Among patients with haematogenous recurrence, the most common site was the liver (66.7%), followed by the lymph nodes, bone, and lung (20.0%, 6.7%, and 3.3%, respectively) (Figures 1A,B).

Compared to local recurrence (median overall survival: 12.4 months, 95% CI, 9.2–15.6) and peritoneal recurrence (median overall survival: 11.3 months, 95% CI, 10.3–12.2), patients with hematogenous recurrence had better prognosis, with median overall survival of 16.9 months (95% CI, 12.5–21.3) (Table 3).

**Table 3:** The prognosis of gastric cancer patients with recurrence (*n* = 122).

**Patterns of recurrence**	**Number of patients**	**Median OS, months**	**95% CI**
Local recurrence	8.2% (12/122)	12.4	(9.2, 15.6)
Hematogenous recurrence	49.2% (58/122)	16.9	(12.5, 21.3)
Liver	69.0% (40/58)	17.6	(15.0, 20.2)
Other sites	31.0% (18/58)	11.7	(9.5, 13.9)
Peritoneal recurrence	42.6% (52/122)	11.3	(10.3, 12.2)
All patients	100% (122/122)	12.2	(11.5, 12.9)

*OS, overall survival; CI, confident interval*.

### Prognostic Significance of Serum Prealbumin and PNI

**Figure 2:**
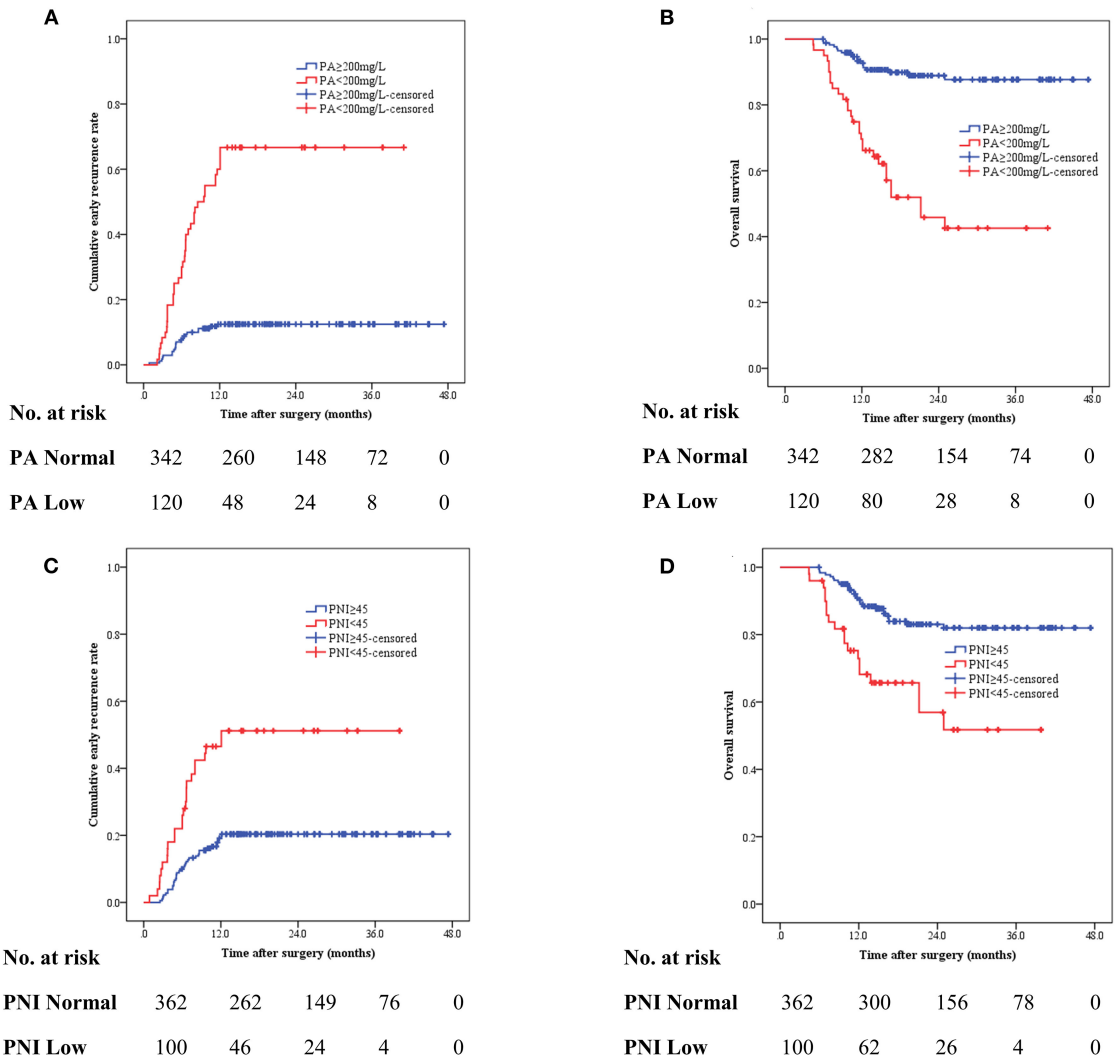
The cumulative recurrence rate and overall survival (OS) in gastric cancer patients based on preoperative serum prealbumin level **(A,B)** and preoperative PNI (<45 vs. ≥45) **(C,D)**.

We investigated the correlation between preoperative nutritional status and the risk of recurrence. As shown in Figure 2A, the recurrence rate was significantly higher in patients with decreased serum prealbumin levels (< 200 mg/L) than in their healthy counterparts (66.7 vs. 12.3%, *p* < 0.001). Meanwhile, OS was significantly shorter among patients with decreased prealbumin levels than among those with normal prealbumin levels at 1 year (70.5 vs. 93.4%) and 3 years (46.3 vs. 88.4%) (Figure 2B). Similarly, compared to those with a normal PNI (50.0 vs. 19.9%, *p* < 0.001), patients with a lower PNI (< 45) had a significantly higher rate of recurrence (Figure 2C). A lower PNI (< 45) was a poor prognostic factor for the 1-year and 3-year OS (Figure 2D).

### Predictive Factors Associated With Recurrence of Gastric Cancer

The predictive factors for postoperative recurrence were identified using univariate analysis (Table 4). The results revealed that gastric cancer recurrence was related to alcohol consumption [yes vs. no, hazard ratio (HR) =2.47, *p* = 0.05], decreased serum albumin (< 35 g/L, HR = 2.03, *p* = 0.009), decreased serum prealbumin (< 200 mg/L, HR = 7.44, *p* < 0.001), and decreased hemoglobin (< 110 g/L, HR = 2.09. *p* = 0.001), lower PNI (< 45, HR = 3.19, *p* < 0.001), serum CEA (≥5 ng/ml, HR = 2.13, *p* = 0.001), serum CA19-9 (≥37 IU/ml, HR = 4.53, *p* < 0.001), tumor size (≥5 cm, HR = 1.56, *p* = 0.015), advanced TNM stage (stage III, HR = 2.27, *p* < 0.001), number of lymph node metastases (≥5, HR = 3.57, *p* < 0.001), signet-ring cell carcinoma (HR = 2.04, *p* = 0.001), tumor deposits (HR = 2.40, *p* < 0.001), vessel carcinoma emboli (HR = 2.26, *p* < 0.001), and adjuvant chemotherapy (HR = 2.98, *p* < 0.001).

**Table 4:** Univariate and multivariate logistic regression analyses of factors associated with recurrence in gastric cancer patients (*n* = 462).

**Variable**	**Univariate**	***p*-value**	**Multivariate**	***p*-value**
	**HR (95%CI)**		**HR (95%CI)**	
Age				
<55	1 (Ref)	0.29	N/A	
≥55	1.22 (0.84–1.76)			
Gender				
Male	1 (Ref)	0.15	N/A	
Female	1.46 (0.87–2.45)			
Body mass index				
≥20	1 (Ref)	0.75	N/A	
<20	1.33 (0.68–3.21)			
Smoking				
No	1 (Ref)	0.13	N/A	
Yes	0.73 (0.48–1.11)			
Alcohol				
No	1 (Ref)	**0.05**	1 (Ref)	0.60
Yes	2.47 (0.98–6.17)		0.83 (0.41–1.68)	
Albumin				
≥35 g/L	1 (Ref)	**0.009**	1 (Ref)	0.58
<35 g/L	2.03 (1.20–3.43)		0.83 (0.43–1.60)	
Prealbumin				
≥200 mg/L	1 (Ref)	**<0.001**	1 (Ref)	**<0.001**
<200 mg/L	7.44 (5.12–10.86)		5.63 (3.67–8.64)	
Preoperative hemoglobin				
≥110 g/L	1 (Ref)	**0.001**	1 (Ref)	0.71
<110 g/L	2.09 (1.47–2.99)		1.09 (0.71–1.65)	
Preoperative serum CEA				
Normal (<5 ng/ml)	1 (Ref)	**0.001**	1 (Ref)	0.28
Elevated (≥5 ng/ml)	2.12 (1.39–3.23)		1.28 (0.94–1.59)	
Preoperative serum CA19-9				
Normal (<37 IU/ml)	1 (Ref)	**<0.001**	1 (Ref)	**<0.001**
Elevated (≥37 IU/ml)	4.53 (2.96–6.93)		2.78 (1.80–4.29)	
PNI				
≥45	1 (Ref)	**<0.001**	1 (Ref)	**0.01**
<45	3.19 (2.23–4.59)		1.73 (1.14–2.63)	
Tumor size				
<5 cm	1 (Ref)	**0.015**	1 (Ref)	0.70
≥5 cm	1.56 (1.09–2.22)		1.09 (0.71–1.66)	
Differentiation				
High/moderate	1 (Ref)	0.133	N/A	
Poor	1.69 (0.88–2.96)			
TNM stage				
II	1 (Ref)	**<0.001**	1 (Ref)	0.40
III	2.27 (1.47–3.50)		1.27 (0.73–2.23)	
Number of lymph node metastasis				
<5	1 (Ref)	**<0.001**	1 (Ref)	**<0.001**
≥5	3.57 (2.49–5.13)		2.48 (1.70–3.61)	
Signet-ring cell carcinoma				
No	1 (Ref)	**0.001**	1 (Ref)	**0.001**
Yes	2.04 (1.34–3.11)		3.26 (2.06–5.16)	
Tumor deposit				
No	1 (Ref)	**<0.001**	1 (Ref)	**0.001**
Yes	2.40 (1.54–3.76)		2.78 (1.72–4.51)	
Vessel carcinoma embolus				
No	1 (Ref)	**<0.001**	1 (Ref)	0.53
Yes	2.26 (1.51–3.38)		0.84 (0.49–1.45)	
Neural invasion				
No	1 (Ref)	0.052	N/A	
Yes	1.64 (0.94–2.45)			
Adjuvant chemotherapy				
Yes	1 (Ref)	**<0.001**	1 (Ref)	**<0.001**
No or incomplete	2.98 (2.01–4.43)		2.82 (1.89–4.22)	
Complication				
No	1 (Ref) 0.314	N/A		
Yes	1.348 (0.78–3.30)			

*For p-value: Boldface type indicates significant difference*.

*HR, hazard ratio; SD, standard deviation; CEA, carcinoembryonic antigen; CA19-9, Carbohydrate Antigen19-9; TNM, tumor-node-metastasis; PNI, prognostic nutritional index; N/A, not applicable; NS, no significant difference*.

Moreover, in the multivariate logistic regression analysis, prealbumin (<200 mg/L), CA19-9 (≥37 IU/ml), PNI (<45), number of lymph node metastasis (≥5), signet-ring cell carcinoma, tumor deposit, and no/incomplete adjuvant chemotherapy were identified as independent predictive factors associated with recurrence of gastric cancer, with the HR of 5.63 (*p* < 0.001, 95% CI 3.67–8.64), 2.78 (*p* < 0.001, 95% CI 1.80–4.29), 1.73 (*p* = 0.010, 95% CI 1.14–2.63), 2.48 (*p* < 0.001, 95% CI 1.70–3.61), 3.26 (*p* = 0.001, 95% CI 2.06–5.16), 2.78 (*p* = 0.001, 95% CI 1.72–4.51) and 2.82 (*p* < 0.001, 95% CI 1.89–4.22), respectively. Details of the univariate and multivariate analyses are presented in Table 4.

## Discussion

### Discussion of the Results

Cancer recurrence after curative gastrectomy is common and is usually associated with poor prognosis. We conducted a large retrospective study to investigate the incidence and patterns of recurrence in patients with stage II/III gastric cancer after laparoscopic D2 gastrectomy. We also evaluated the association between nutritional status and postoperative recurrence, which may provide a prospective tool for preoperative prediction, intervention, and postoperative surveillance of high-risk patients. Our results demonstrated that 26.4% (122/462) of the patients experienced a recurrence within the follow-up period after laparoscopic D2 gastrectomy. The most common recurrence pattern was haematogenous recurrence (49.2%), and the most common site was the liver (66.7%). Furthermore, we found that preoperative nutritional parameters such as serum prealbumin level and PNI were independent predictive factors for postoperative recurrence.

### Patterns and Prognosis of Recurrence

According to the literature, gastric cancer recurrence patterns are usually classified as locoregional, haematogenous, or peritoneal. Locoregional recurrence was defined as cancer recurrence at the resection site or in local lymph nodes. Haematogenous recurrence is defined as a recurrent lesion in distant organs, such as the liver, lung, bone, brain, and ovary. Peritoneal recurrence, with or without multiple sites, was defined as cancer recurrence in the abdominal cavity (7, 21). Based on previous reports, recurrence of gastric cancer after curative surgery usually occurs within 2 years after surgery (7–9, 21, 22). Our study also showed that most recurrences occurred within 2 years after surgery, with a median RFS of 17.5 months. Compared to patients without recurrence, those with recurrence had a poor prognosis (shorter OS). The most common site of haematogenous recurrence was the liver, with an OS of 17.6 months (95% CI, 15.0–20.2) (Table 2). A possible explanation is that systematic chemotherapy or treatments such as liver resection or transcatheter arterial haemoembolization (TACE), might benefit this group of patients.

### Malnutrition Is an Independent Predictive Factor for Recurrence

It has been widely reported that nutritional status affects long-term survival in various cancers. Clinicians use the following tools to evaluate the nutritional status of patients: physical data, weight loss, body mass index (BMI), serum albumin and prealbumin levels, Glasgow prognostic score, controlling nutritional status, and prognostic nutritional index (PNI) (13–16, 23). In our study, we proved that preoperative nutritional status may have an impact on gastric cancer recurrence. In univariate and multivariate analysis, we revealed that both serum prealbumin and PNI were independent predictive factors for recurrence, with an HR of 5.63 (95% CI 3.67–8.64) and 1.73 (95% CI 1.14–2.63), respectively. Furthermore, we explored the differences in the clinical and pathological characteristics between the normal and decreased prealbumin groups. We found that patients with decreased prealbumin levels had an advanced TNM stage (*p* = 0.011) and a higher ratio of lymph node metastasis (*p* = 0.002) (Supplementary Table 1). In the subgroup analysis, patients with decreased prealbumin presented with significantly shorter RFS and OS in both the stage II and stage III groups (Supplementary Figure 2).

Prealbumin, also known as transthyretin, is an acute-phase liver protein with a short half-life of 2–3 days. Prealbumin level reflects liver function and has emerged as an indicator of inflammation. With a relatively short half-life, prealbumin is a more sensitive biomarker than albumin for assessing the nutritional status (24, 25). Furthermore, prealbumin is a predictor of postoperative complications and overall survival in various gastrointestinal malignancies, such as gastric cancer and hepatocellular carcinoma (26, 27). A recent meta-analysis showed that a low pretreatment serum prealbumin level predicts unfavorable prognosis in patients with gastrointestinal cancers (28). The prognostic nutritional index (PNI), which is based on serum albumin and absolute peripheral lymphocyte count, has also been proven to be a prognostic factor for postoperative complications and long-term survival (23, 29). In the present study, we evaluated the association between preoperative nutritional status and recurrence of stage II/III gastric cancer after D2 gastrectomy. Our results showed that these easily accessible nutritional markers, such as prealbumin and PNI, can be used to predict the recurrence of gastric cancer. The underlying reason for this association is that patients with gastric cancer who suffer from malnutrition may have impaired immune functions. Since the immune system plays a crucial role in carcinogenesis and cancer metastasis, impaired immune function may lead to cancer recurrence and increased mortality by disturbing T-cell metabolism or other mechanisms (30, 31).

### Clinicopathological Predictive Factors for Gastric Cancer Recurrence

Numerous studies have revealed that clinicopathological characteristics, such as tumor site, increased CEA and CA19-9 levels, increased neutrophil-lymphocyte ratio (NLR), advanced TNM stage, lymph node ratio, tumor size, poor cell differentiation, tumor deposit, signet-ring cell, neural and lymphatic invasion, and incomplete chemotherapy, are risk factors for poor prognosis in gastric cancer (32–36). Predictive models were constructed to discriminate between patients with a high risk of postoperative recurrence (37). Ghidini et al. identified poor prognostic factors for overall survival in gastric cancer in a recent, large-scale study. The factors included older age, presence of cardiac tumors, lack of postoperative chemotherapy, positive resection margins, and advanced stages (38). In our study, we found that elevated CA19-9 levels, increased the number of lymph node metastases, presence of tumor deposits, presence of signet-ring cell carcinoma, and incomplete adjuvant chemotherapy were independent predictive factors for the recurrence of stage II/III gastric cancer after laparoscopic D2 gastrectomy. These findings are consistent with those of previous studies. Since prealbumin, PNI, and adjuvant chemotherapy are factors that can be managed, timely intervention may potentially reduce the risk of cancer recurrence.

### Clinical Implication and Limitations

Patients with gastric cancer who experience recurrence may have a poor prognosis. Although most studies regarding liver or local recurrence of gastric cancer were retrospective and from a single institution, researchers have found that patients receiving further treatments, such as surgery or systemic chemotherapy, generally survived longer (39–41). Therefore, identifying predictive factors for recurrence is important and can impact a patient's comprehensive treatment and follow-up plans. We proved that preoperative malnutrition contributed to postoperative recurrence of stage II/III gastric cancer. Preoperative nutritional support may benefit these patients and improve their overall outcome. The purpose of follow-up is to identify patients with recurrence for further intervention. Our study identified seven independent predictive factors contributing to recurrence after curative laparoscopic D2 gastrectomy, and helped doctors identify patients with predictive factors for intense monitoring after surgery.

The present study has several limitations that should be considered. First, in this retrospective setting, the amount of data regarding which factors might contribute to recurrence is small and might be incomplete. Furthermore, pathological factors that may influence serum prealbumin levels, such as hepatitis, liver cirrhosis, and chronic nephritis, were not recorded in this study. Second, this study had a relatively small sample size from a single institution without external validation. Third, differences exist in the outcomes of gastric cancer between the eastern and western populations. The difference in long-term survival may be affected by factors such as eating habits, the incidence rate of *Helicobacter pylori* (HP) infection, the detection rate of early gastric cancer, and standard surgical procedures (42, 43). The conclusion of this study may not be conclusive for the Western population. Fourth, information on the treatment plans for patients with recurrence is incomplete. Therefore, a prospective study with a larger sample size and more clinicopathological measurements is needed to validate the findings of our study and to explore more potential predictive factors for the recurrence of gastric cancer.

## Conclusions

In summary, our study is the first to indicate that nutritional status, such as prealbumin level and PNI, is significantly associated with recurrence in patients with gastric cancer receiving laparoscopic D2 gastrectomy. Second, the preoperative nutritional status can be easily evaluated and applied to identify patients with malnutrition for nutritional support and intensive follow-up.

## Data Availability Statement

The raw data supporting the conclusions of this article will be made available by the authors, without undue reservation.

## Ethics Statement

This study was approved by the Institutional Medical Ethics Committee (TJH20190901), with all aspects in this study complying with the 1964 Helsinki Declaration and later versions. Informed consent was obtained from all patients included.

## Author Contributions

SZ and CG: conceptualization and writing the original draft. YT: data curation, methodology, and software. CD and LZ: formal analysis and project administration. All authors participated in the study design and have agreed to the final version and meet the major criteria recommended by the ICMJE (http://www.icmje.org/). All authors contributed to the article and approved the submitted version.

## Funding

This study was supported by grants from the Chinese Society of Clinical Oncology (no. Y-sy2018-029).

## Conflict of Interest

The authors declare that the research was conducted in the absence of any commercial or financial relationships that could be construed as a potential conflict of interest.

## Publisher's Note

All claims expressed in this article are solely those of the authors and do not necessarily represent those of their affiliated organizations, or those of the publisher, the editors and the reviewers. Any product that may be evaluated in this article, or claim that may be made by its manufacturer, is not guaranteed or endorsed by the publisher.

## Supplementary Material

Supplementary Figure 1 The flow chart of the included patients.

Supplementary Figure 2 Recurrence-free survival (RFS) and overall survival (OS) in stage II **(A,C)** and stage III **(B,D)** gastric cancer patients based on preoperative serum prealbumin (PA) levels.

Supplementary Table 1 Clinical characteristics of patients with normal or decreased prealbumin levels (*n* = 462).
