# Supplementary figures and images for: Preoperative Low Prealbumin Is Associated With Recurrence in Patients With Stage II/III Gastric Cancer After Laparoscopic D2 Gastrectomy
Source: Front Surg. 2022 Apr 1;9:819514. doi: 10.3389/fsurg.2022.819514 (PMC9010530; doi:10.3389/fsurg.2022.819514)

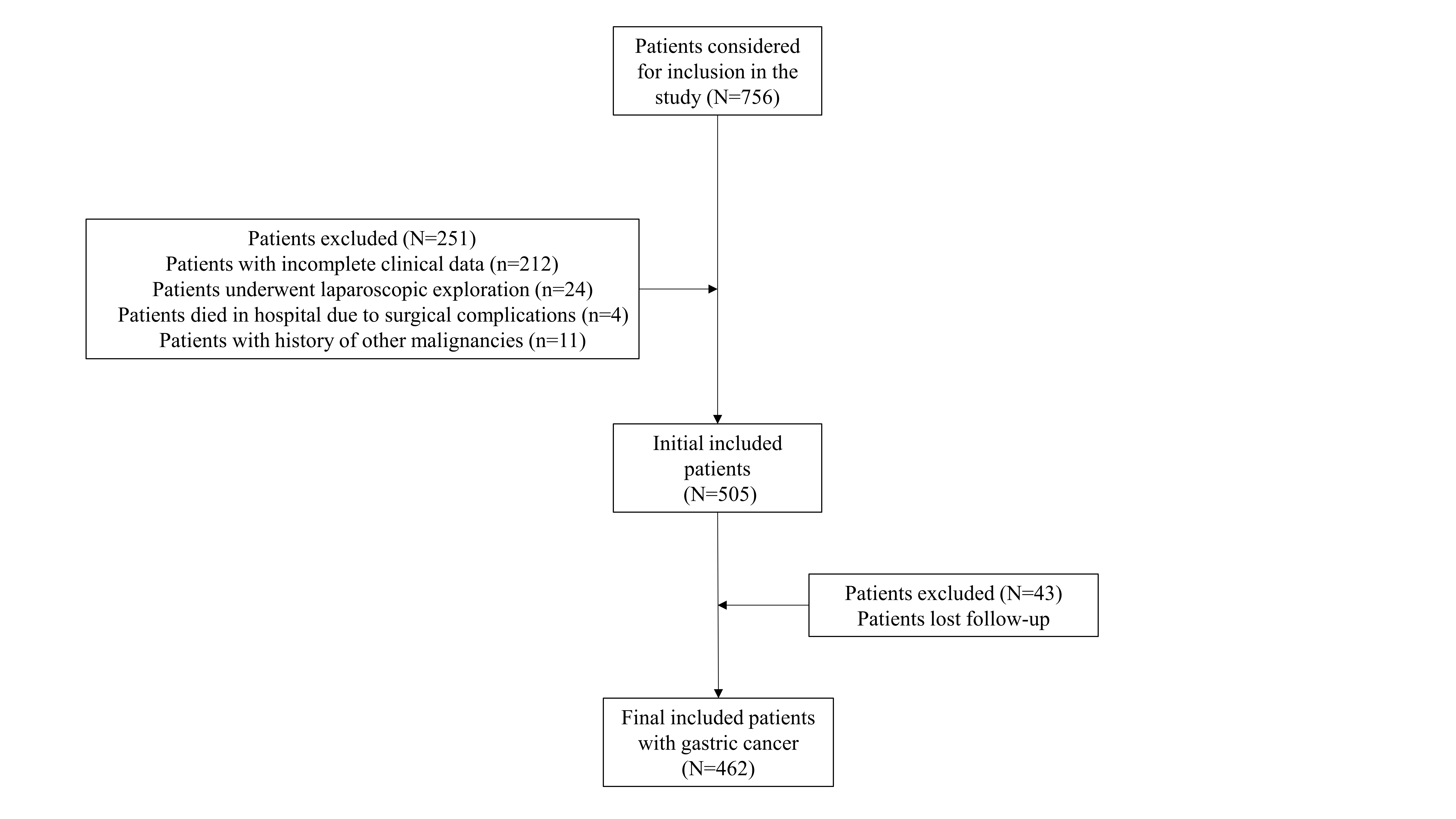

Supplement: Supplementary Figure 1 — The flow chart of the included patients. [file Image_1.TIF]

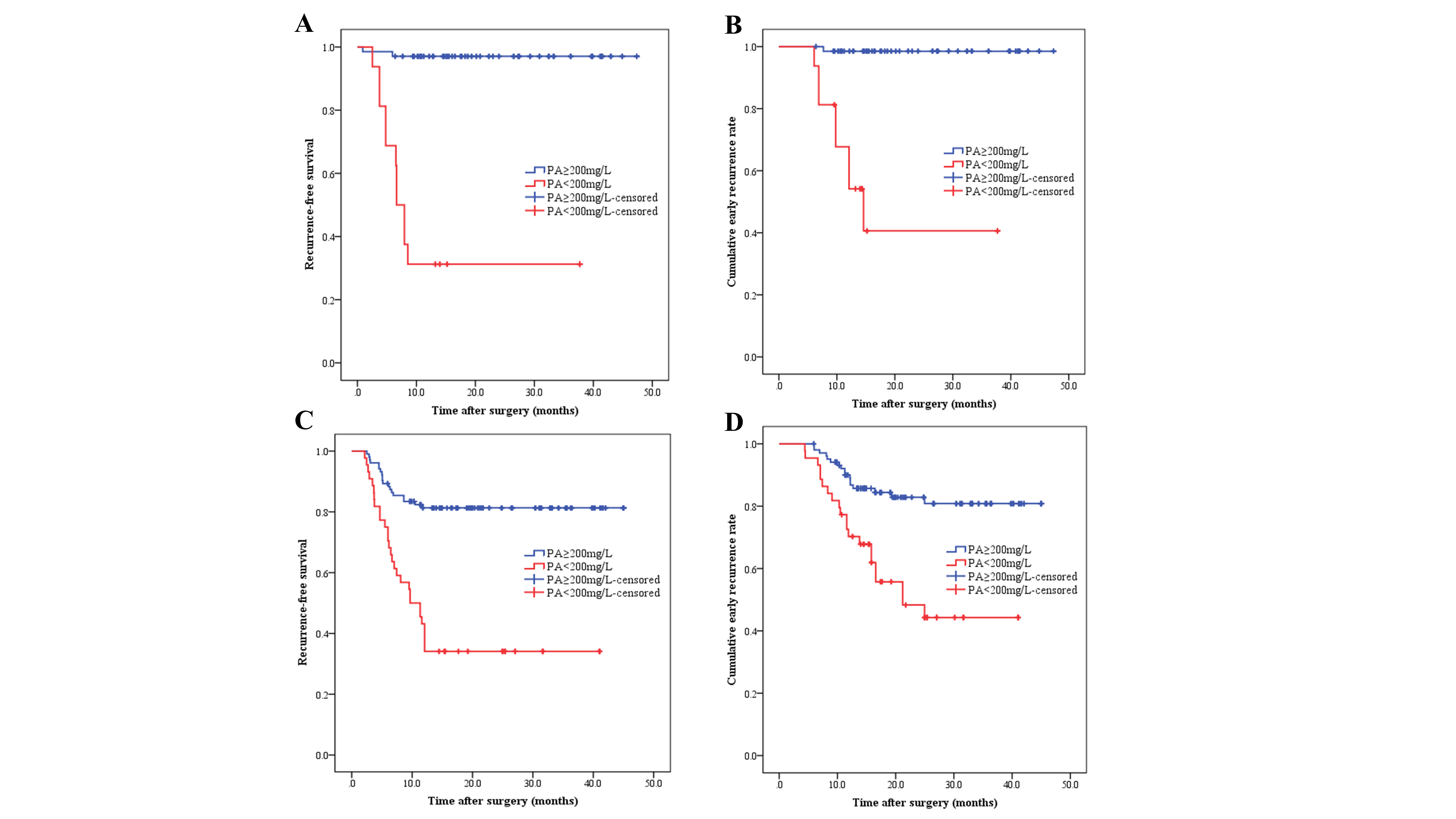

Supplement: Supplementary Figure 2 — Recurrence-free survival (RFS) and overall survival (OS) in stage II (A,C) and stage III (B,D) gastric cancer patients based on preoperative serum prealbumin (PA) levels. [file Image_2.TIF]
