# Supplementary material for: Preoperative Low Prealbumin Is Associated With Recurrence in Patients With Stage II/III Gastric Cancer After Laparoscopic D2 Gastrectomy
Source: Front Surg. 2022 Apr 1;9:819514. doi: 10.3389/fsurg.2022.819514 (PMC9010530; doi:10.3389/fsurg.2022.819514)
Supplement: Supplementary Table 1 — Clinical characteristics of patients with normal or decreased prealbumin levels (n = 462). [file Table_1.docx]

Supplemental Table 1. The clinical characteristics in patients with normal and decreased prealbumin (n=462)

| **Variables** | **Normal prealbumin (n=342)** | **Decreased prealbumin (n=120)** | **p-value** |
| --- | --- | --- | --- |
| TNM stage  II  III | 39.8% (136/342)  60.2% (206/342) | 26.7% (32/120)  73.3% (88/120) | **0.011** |
| N stage  0  1  2  3 | 35.1% (120/342)  22.8% (78/342)  23.4% (80/342)  18.7% (64/342) | 10.0% (12/120) 、25.0% (30/120) 33.3% (40/120) 31.7% (38/120) | **0.002** |
| Signet-ring cell carcinoma  No  Yes | 86.0% (294/342)  14.0% (48/342) | 83.3% (100/120)  16.7% (20/120) | 0.549 |
| Tumor deposit  No  Yes | 88.3% (302/342)  11.7% (40/342) | 91.7% (110/120)  8.3% (10/120) | 0.393 |
| Vessel carcinoma embolus  No  Yes | 97.7% (334/342)  2.3% (8/342) | 96.7% (116/120)  3.3% (4/120) | 0.519 |

For p-value: Boldface type indicates significant difference.

SD, standard deviation; TNM, tumor-node-metastasis.
